# Interstock-Mediated Graft Incompatibility: Insights into Photosynthetic Pigments, Carbohydrates, Antioxidant Defense Systems, and Hormones Response Mechanisms in Citrus

**DOI:** 10.3390/plants14040522

**Published:** 2025-02-08

**Authors:** Tie Wang, Zhenghua Jin, Ya Yuan, Lijun Deng, Guochao Sun, Siya He, Ling Liao, Jun Wang, Bo Xiong, Zhihui Wang

**Affiliations:** College of Horticulture, Sichuan Agricultural University, Chengdu 611130, China

**Keywords:** top grafting, interstock, photosynthetic pigment, enzyme activity, phytohormone

## Abstract

Interstock, located between rootstock and scion, plays a critical role in determining graft compatibility. This study aimed to elucidate the physiological mechanisms mediated by interstock in graft compatibility by comparing various leaf and root system parameters between compatible and incompatible graft combinations. These parameters included growth parameters, photosynthetic pigments, carbohydrates, antioxidant enzyme systems, and hormones. The study found that both PG (‘Yuanxiaochun’/‘Ponkan’/‘*Trifoliate orange*’) and JJ (‘Yuanxiaochun’/‘Kumquat’/‘*Trifoliate orange*’) treatments exhibited a noticeable phenomenon of “small feet” (scion diameter exceeding interstock), indicating mild graft incompatibility. Compared to grafted compatibility groups, chlorophyll content in PG and JJ treatments leaves was significantly reduced, particularly in carotenoids (Car). Additionally, PG and JJ treatments leaves showed lower levels of total soluble sugars, fructose, sucrose, gibberellin A4, zeatin-Riboside, and N6-(delta2-isopentenyl) adenosine, as well as catalase (CAT) activity. In contrast, peroxidase (POD) activity, glucose, soluble proteins, hydrogen peroxide (H_2_O_2_), malondialdehyde (MDA), aminocyclopropane carboxylic acid, and abscisic acid content were higher. In roots, PG and JJ treatments had elevated starch, sucrose, jasmonic acid, and jasmonic acid-isoleucine content, but showed lower levels of total soluble sugars, MDA, indole-3-acetic acid, and abscisic acid. Comprehensive analysis revealed that total soluble sugar content in both leaves and roots under PG and JJ treatments were reduced. These findings offer valuable insights into enhancing citrus grafting practices, particularly by guiding the selection of compatible rootstock-scion combinations. By elucidating the physiological mechanisms underlying graft compatibility, this research enables researchers and growers to refine grafting strategies, thereby improving citrus grafting success rates.

## 1. Introduction

The technique of grafting, a time-honored approach to enhance plant propagation, has been employed for more than two millennia [1,2]. Prior studies have demonstrated that appropriate grafting combinations possess the potential to enhance the tolerance of grafting combinations [3]. Moreover, they also exert a positive influence on plant growth [4], stimulating early flowering in the scion [5], and enhancing the overall fruit quality [6]. Nonetheless, it has been observed in certain investigations that grafting combinations that are not well-suited can result in the inhibition of scion growth, leaf yellowing, subsequently followed by a reddish or orange hue, early defoliation, leaf dieback, and an earlier halt to tree growth [7]. Consequently, the meticulous matching and careful selection of rootstock and scion combinations assumes paramount significance.

Citrus, being the leading fruit industry globally [8], holds significant significance in economic development and the well-being of individuals. The remarkable growth of the citrus industry can be attributed to the advancement and refinement of grafting technology. Nonetheless, the continuous introduction of novel citrus varieties has led to the prevailing practice of top grafting on existing citrus trees for rapid variety renewal [9]. This approach offers notable benefits such as effectiveness and expedited production [10]. In our previous study, it was demonstrated that the utilization of top grafting propagation technique has become widespread within the citrus industry in Sichuan Province, China [9]. Interestingly, it was observed that ‘Yuanxiaochun’ ((*Citrus unshiu* Marcov × *Citrus sinensis* Osbeck) × (*Citrus reticulata* × *Citrus paradisi*)) with ‘Ponkan’ (*Citrus reticulata* Blanco cv. Ponkan) and ‘Kumquat’ (*Fortunella margarita* Lour. Swingle) as the interstock exhibited noticeable “small feet” (scion diameter exceeding interstock) [11]. However, the precise mechanism behind its formation remains unreported.

“Small feet” is considered a mild form of incompatibility in graft compatibility assessment [12]. The physiological mechanisms behind grafting, including compatibility factors, callus formation, healing processes, and the signaling interactions between the scion and rootstock, remain poorly understood. It is unclear whether grafting incompatibility arises from rejection of the graft partners, differences in growth rates, or the stress caused by the grafting procedure itself [1]. In order to scientifically assess graft-induced incompatibility, researchers have established various evaluation methods. For example, Mosse et al. [13] classified grafting incompatibility into two types: ‘Translocated’ and ‘Localized’ incompatibility. Characteristics of ‘Translocated’ incompatibility can be visually identified, such as leaf yellowing, cessation of tree growth, or halted root development. On the other hand, ‘Localized’ incompatibility is characterized by anatomical abnormalities observed at the graft union after dissection. Although this evaluation method is relatively simple, it carries the risk of subjective influence. As a result, researchers have also conducted studies from a physiological perspective to address this issue. For example, grafting ‘Hongmianmiyou’/‘*Trifoliate orange*’ in citrus resulted in pronounced leaf yellowing after five months, further revealing significantly higher starch content in these leaves, with changes in the plant hormones indole-3-acetic acid and abscisic acid identified as major components [14]. Research evaluating graft compatibility in Rimau Gerga Lebong (RGL) found that the RGL/Japansche citroen combination exhibited late incompatibility, characterized by lower graft survival rates, growth parameters, and chlorophyll indices [15]. In peach and plum studies, it was found that grafting incompatible combinations resulted in increased starch content in scion stems, as well as significantly enhanced levels of total soluble sugars, phenolic compounds, and antioxidant enzyme activity [16]. Molecular methods have also been applied in graft incompatibility research. For example, He et al. [17] found that the yellowing of leaves caused by grafting incompatibility in pomelo was significantly positively correlated with high expression of *CgGBSS2*, and gene functional validation confirmed this conclusion. In addition, researchers have identified a set of genes that influence graft compatibility, such as candidate genes *SlWOX4*, *GH9B3*, and *THOM1* [18].

Currently, there is considerable research on rootstock-mediated graft incompatibility, but there have been no reports on interstock-mediated graft incompatibility in citrus. To delve into the physiological mechanisms underlying graft-induced “small feet”, this study systematically investigates growth parameters, chlorophyll content, carbohydrates, antioxidant enzymes, osmotic regulators, and hormone types and levels in grafted combinations showing normal growth versus those exhibiting “small feet”. Additionally, correlation analysis was employed to identify key indicators associated with the occurrence of “small feet”. These research findings aim to enhance our understanding of “small feet” phenomenon and provide guidance for advanced grafting practices in the industry.

## 2. Results

### 2.1. Effects of Different Interstocks on the Growth of Trees

**Figure 1:**
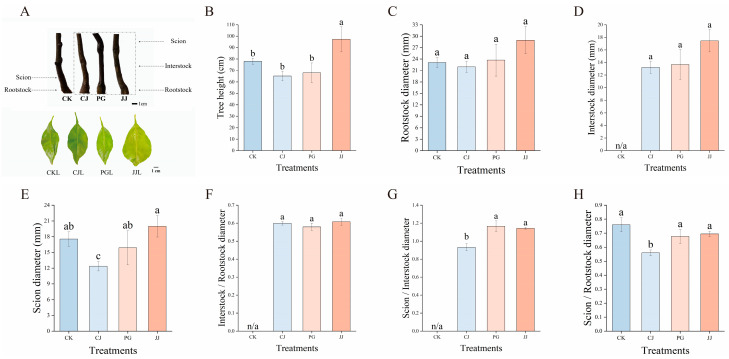
The effect of interstocks on tree growth. (**A**) Rootstock and leaf morphology of different grafting treatments. (**B**) Tree height. (**C**) Rootstock diameter. (**D**) Interstock diameter. (**E**) Scion diameter. (**F**) Interstock/rootstock diameter. (**G**) Scion/interstock diameter. (**H**) Scion/rootstock diameter. The bars from left to right represent the different treatments, CK, CJ, PG and JJ. ‘n/a’ indicates not applicable. The parameter values presented in each figure are indicated as mean ± standard deviation (SD) (*n* = 3), different letters denote statistically differences between different treatments (Tukey’s test, *p* < 0.05).

Based on Figure 1A, PG and JJ exhibited a “small feet” phenotype, while CK and CJ appeared normal. Further observation of leaf characteristics in different grafting combinations revealed that grafted combinations with the “small feet” phenotype exhibited significant leaf yellowing [13]. In terms of tree height, JJ treatment was significantly the tallest at 97.33 cm, while the other three treatments showed no significant differences (Figure 1B). Although rootstock and interstock diameter differences across various grafting treatments were not significant, the JJ treatment consistently had the largest values, measuring 28.96 mm and 17.47 mm, respectively (Figure 1C,D). Regarding scion diameter, the JJ treatment showed the largest diameter at 19.98 mm, while the CJ treatment had the smallest diameter at 12.38 mm (Figure 1E). In addition, the ratios of interstock diameter to rootstock diameter, and scion diameter to rootstock diameter were all less than 1 across all treatments. However, in the ratio of scion diameter to interstock diameter, CJ treatment was less than 1, while PG and JJ treatments were both greater than 1 (1.17 and 1.14, respectively), indicating a pronounced phenomenon of “small feet” (Figure 1F–H). Comprehensive analysis indicates that different interstocks exert varying effects on the growth of ‘Yuanxiaochun’ trees. Notably, PG and JJ treatments exhibited a pronounced “small feet” phenotype accompanied by significant leaf yellowing, suggesting the presence of mild graft incompatibility characteristics [12].

### 2.2. Effects of Different Interstocks on Leaf Chlorophyll of ‘Yuanxiaochun’

**Figure 2:**
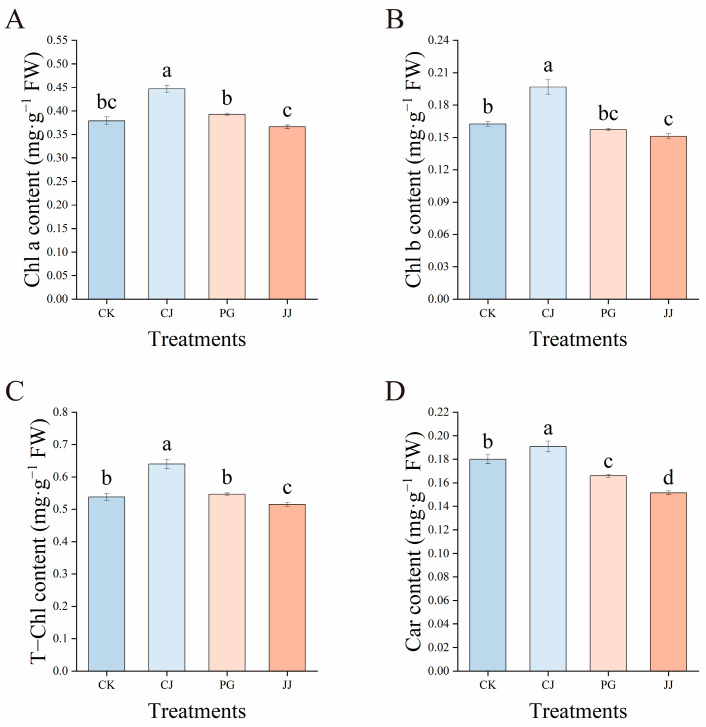
Effects of different interstocks on the chlorophyll content of ‘Yuanxiaochun’ leaves. (**A**) Chl a. (**B**) Chl b. (**C**) T-Chl. (**D**) Car. The bars from left to right represent the different treatments, CK, CJ, PG and JJ. The parameter values presented in each figure are indicated as mean ± standard deviation (SD) (*n* = 3), different letters denote statistically differences between different treatments (Tukey’s test, *p* < 0.05).

To assess the impact of interstock on the chlorophyll content of ‘Yuanxiaochun’ leaves, the study measured and analyzed chlorophyll (Chl) content in leaves of different grafting combinations (Figure 2). CJ treatment significantly increased the contents of Chl a, Chl b, T-Chl, and Car in leaves, while JJ treatment resulted in relatively lower levels. In terms of Chl a, Chl b, and T-Chl content, there were no significant differences between CK and PG treatments; however, Car content in PG treatment was significantly lower, at 92.22% of CK treatment. Further comprehensive analysis of the data revealed that both PG and JJ treatments with “small feet” characteristics had significantly lower leaf Car content, indicating that this characteristic inhibited Car accumulation in ‘Yuanxiaochun’ leaves.

### 2.3. Effect of Different Interstocks on Tree Leaf and Root Carbohydrates

#### 2.3.1. Starch and Soluble Sugar

**Figure 3:**
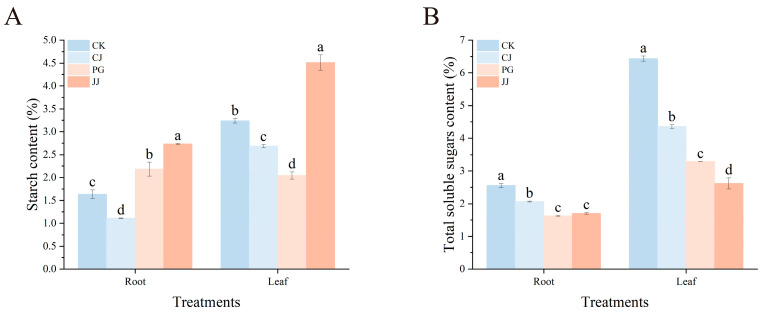
Impact of different interstocks on leaf and root starch, and total soluble sugar content in trees. (**A**) Starch. (**B**) Total soluble sugar. The bars from left to right represent the different treatments, CK, CJ, PG and JJ. The parameter values presented in each figure are indicated as mean ± standard deviation (SD) (*n* = 3), different letters denote statistically differences between different treatments (Tukey’s test, *p* < 0.05).

To investigate the effect of interstock on tree carbohydrate content, we measured starch and total soluble sugar content in leaves and roots of trees with different grafting combinations (Figure 3). The study found that JJ treatment showed significantly higher starch content in both leaves and roots, measuring 4.51% and 2.73%, respectively. In contrast, PG treatment showed significantly lower starch content in leaves at 2.04%. Total soluble sugar content in leaves and roots of CK treatment was significantly higher at 6.44% and 2.56%, respectively. Conversely, JJ treatment showed the lowest values, at only 2.62% and 1.70%. In addition, the results showed that total soluble sugar content in leaves and roots of CK and CJ treatments was significantly higher, while PG and JJ treatments, which exhibited the “small feet” characteristic, showed significantly lower levels. Overall, the analysis indicates that PG and JJ treatments resulted in lower total soluble sugar content in leaves and roots of tree.

#### 2.3.2. Sugar Components

**Figure 4:**
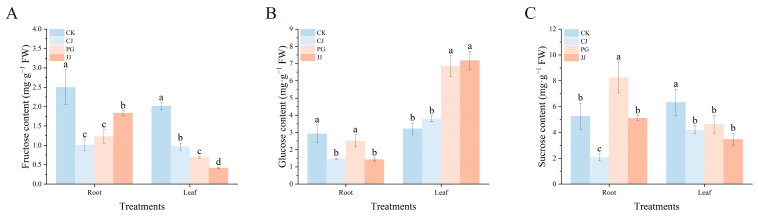
Effect of different interstocks on sugar components of tree leaves and roots. (**A**) Fructose. (**B**) Glucose. (**C**) Sucrose. The bars from left to right represent the different treatments, CK, CJ, PG and JJ. The parameter values presented in each figure are indicated as mean ± standard deviation (SD) (*n* = 3), different letters denote statistically differences between different treatments (Tukey’s test, *p* < 0.05).

Further analysis of sugar components in leaves and roots of different grafting combinations revealed that CK and CJ treatments had relatively high fructose content in leaves, while PG and JJ treatments showed lower levels (Figure 4A). For glucose content, leaves from JJ and PG treatments had significantly higher levels, measuring 7.17 mg·g^−1^ FW and 6.85 mg·g^−1^ FW, respectively (Figure 4B). In addition, CK treatment had the highest sucrose content in leaves at 6.32 mg·g^−1^ FW (Figure 4C).

At the roots, CK showed higher levels of all three sugars, while CJ showed the opposite trend. PG treated roots had the highest glucose and sucrose content at 2.51 mg·g^−1^ FW and 8.25 mg·g^−1^ FW, respectively (Figure 4). Overall, leaves from PG and JJ treatments (which exhibited the characteristic “small feet”), had lower fructose and higher glucose content, suggesting that this may be related to the response of ‘Yuanxiaochun’ leaves to the “small feet” trait. A comparison of fructose, sucrose, and total soluble sugar content in the leaves indicated a consistent trend, demonstrating that low levels of fructose and sucrose were directly responsible for the reduced total soluble sugar content in the leaves of the PG and JJ treatments (Figure 3B and Figure 4A).

### 2.4. Effects of Different Interstocks on Antioxidant Enzymes, Osmoregulatory Substances, H_2_O_2_ and MDA in Leaf and Root of Trees

#### 2.4.1. Antioxidant Enzyme

**Figure 5:**
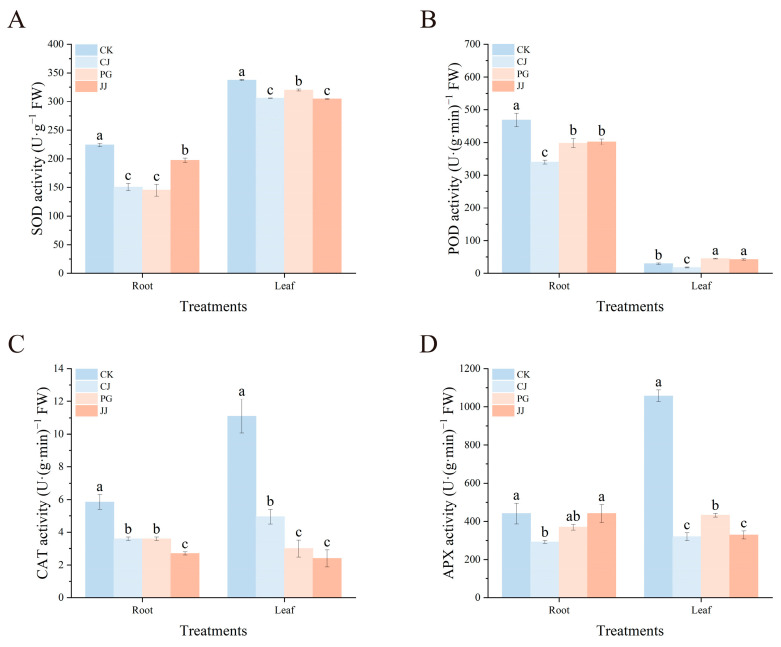
Effects of different interstocks on antioxidant enzyme activity of tree leaves and roots. (**A**) SOD. (**B**) POD. (**C**) CAT. (**D**) APX. The bars from left to right represent the different treatments, CK, CJ, PG and JJ. The parameter values presented in each figure are indicated as mean ± standard deviation (SD) (*n* = 3), different letters denote statistically differences between different treatments (Tukey’s test, *p* < 0.05).

To investigate the effect of interstock on the antioxidant enzyme system in roots and leaves of grafted trees, the activities of SOD, POD, CAT, and APX were measured and analyzed. The results showed that SOD, CAT, and APX activities were generally higher in leaves than in roots, while POD activity was significantly higher in roots than in leaves (Figure 5). Among the four treatments, JJ treatment had relatively lower leaf SOD, CAT, and APX activity, while CK treatment showed the opposite trend. In addition, POD activity in leaves of PG and JJ treatments was significantly higher than that of CK and CJ treatments.

At root, POD activity trends in the other three treatments, excluding CK, were consistent with those in leaves, with JJ showing the highest activity and CJ the lowest at 401.85 U·(g·min)^−1^ FW and 340.20 U·(g·min)^−1^ FW, respectively. Notably, although CK treatment had lower POD activity in leaves, its root activity was significantly higher, measuring 468.60 U·(g·min)^−1^ FW. Overall, leaves from PG and JJ treatments exhibited significantly higher POD activity. This suggests that the interstock-mediated “small feet” trait promotes POD accumulation in scion leaves while simultaneously reducing CAT activity.

#### 2.4.2. Osmoregulatory Substances, H_2_O_2_ and MDA

**Figure 6:**
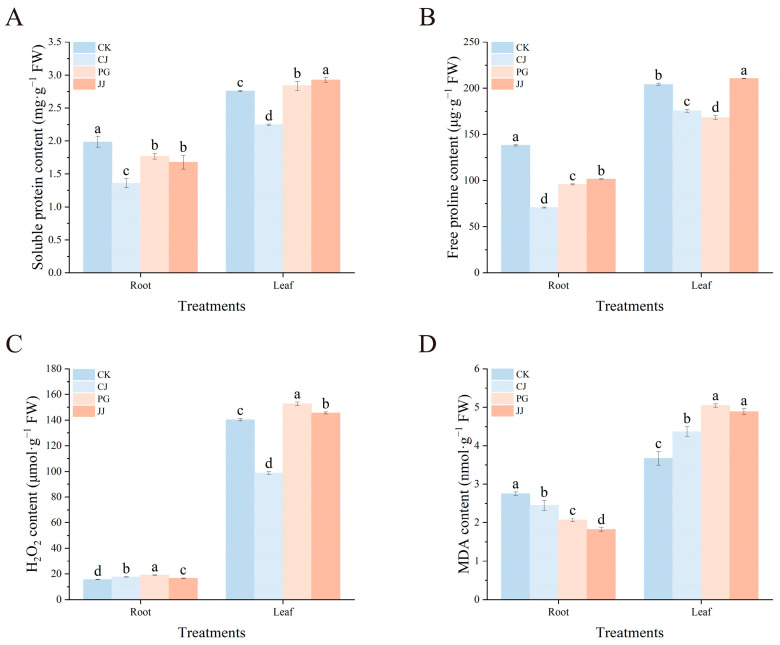
Effects of different interstocks on osmoregulatory substances, H_2_O_2_ and MDA of tree leaves and roots. (**A**) Soluble protein, (**B**) Free proline. (**C**) H_2_O_2_. (**D**) MDA. The bars from left to right represent the different treatments, CK, CJ, PG and JJ. The parameter values presented in each figure are indicated as mean ± standard deviation (SD) (*n* = 3), different letters denote statistically differences between different treatments (Tukey’s test, *p* < 0.05).

Based on Figure 6A,B, soluble protein and free proline levels were notably lower in roots than leaves across all treatments. Regarding soluble protein content, CJ treatment exhibited significantly lower levels in roots and leaves, at 1.36 mg·g^−1^ FW and 2.25 mg·g^−1^ FW, respectively. Free proline levels were lowest in CJ roots and PG leaves, at 70.79 μg·g^−1^ FW and 168.43 μg·g^−1^ FW, respectively. Moreover, JJ treatment showed significantly higher levels of soluble protein and free proline in leaves compared to other treatments. Further findings revealed lower H_2_O_2_ content in CJ leaves and CK roots, at 98.87 μmol·g^−1^ FW and 15.59 μmol·g^−1^ FW, respectively. It is noteworthy that PG and JJ treatments exhibited higher H_2_O_2_ content in both leaves and roots (Figure 6C). In terms of MDA content, MDA levels in leaves of PG and JJ were significantly higher than those in leaves of CK and CJ. The MDA content in roots treated with JJ was significantly lower at 1.82 nmol·g^−1^ FW. The above results indicated that both H_2_O_2_ and MDA were significantly enriched in the scion leaves of grafting combinations characterized by “small feet” (Figure 6D).

### 2.5. Effect of Different Interstocks on Leaf and Root Hormones of Trees

To clarify the effects of interstock on the types and contents of hormones in the roots and leaves of grafted trees, the experiment employed liquid chromatography coupled with high-resolution mass spectrometry to detect the hormones in the leaves and roots of different grafting combinations. Based on the roles of hormones in regulating plant growth and development, this study classifies hormones into categories such as growth-promoting hormones, growth-inhibiting hormones, and other hormones, following the classification standards established by previous researchers [19].

#### 2.5.1. Growth-Promoting Hormone

**Figure 7:**
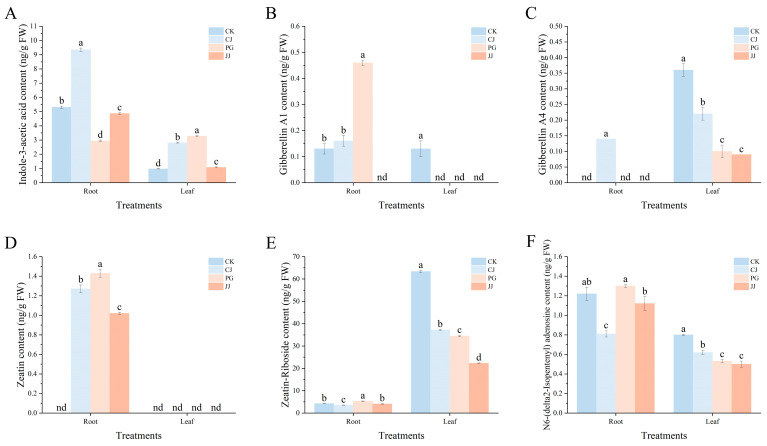
Effects of different interstocks on the growth−promoting hormone content of tree leaves and roots. (**A**) Indole-3-acetic acid. (**B**) Gibberellin A1. (**C**) Gibberellin A4. (**D**) Zeatin. (**E**) Zeatin-Riboside. (**F**) N6-(delta2-Isopentenyl) adenosine. ‘nd’ indicates not detected. The parameter values presented in each figure are indicated as mean ± standard deviation (SD) (*n* = 3), different letters denote statistically differences between different treatments (Tukey’s test, *p* < 0.05).

In this experiment, a total of 6 growth-promoting hormones were detected. Among them, indole-3-acetic acid, zeatin-Riboside, and N6-(delta2-Isopentenyl) adenosine were detected in both root and leaf, while zeatin was detected only in root. Gibberellin A1 or gibberellin A4 were detected simultaneously in root and leaf of only some grafting combinations (Figure 7). In leaf hormone content, gibberellin A4, zeatin-Riboside, and N6-(delta2-Isopentenyl) adenosine showed a similar pattern, as demonstrated by significantly higher CK and CJ treatments than PG and JJ treatments. In root, gibberellin A4 was detected only in CJ root, at a concentration of 0.14 ng·g^−1^ FW. PG treatment showed significantly higher levels of gibberellin A1, zeatin-Riboside, and N6-(delta2-Isopentenyl) adenosine compared to other treatments, while indole-3-acetic acid content was significantly the lowest at 2.94 ng·g^−1^ FW. In the zeatin content, PG had the highest content, whereas JJ had the lowest, at 1.43 ng·g^−1^ FW and 1.02 ng·g^−1^ FW, respectively. Comprehensive analysis indicates that different interstocks significantly affect hormone levels in the roots and leaves of grafted combinations. Specifically, PG and JJ treatments, characterized by the “small feet” trait, resulted in lower levels of gibberellin A4, zeatin-Riboside, and N6-(delta2-Isopentenyl) adenosine in the leaves of ‘Yuanxiaochun’.

#### 2.5.2. Growth-Inhibiting Hormone

**Figure 8:**
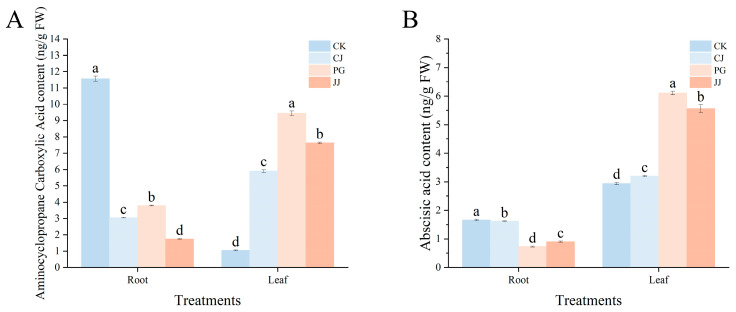
Effects of different interstocks on the growth−inhibiting hormone content of ‘Yuanxiaochun’ leaf and root. (**A**) Aminocyclopropane carboxylic acid. (**B**) Abscisic acid. The parameter values presented in each figure are indicated as mean ± standard deviation (SD) (*n* = 3), different letters denote statistically differences between different treatments (Tukey’s test, *p* < 0.05).

In this study, the aminocyclopropane carboxylic acid content in PG and JJ leaves was significantly higher than other treatments at 9.45 ng·g^−1^ FW and 7.63 ng·g^−1^ FW, respectively. In root, CK had the highest aminocyclopropane carboxylic acid content at 11.57 ng·g^−1^ FW (Figure 8A). The abscisic acid content in leaves showed a similar trend to aminocyclopropane carboxylic acid, where PG and JJ had significantly higher levels at 6.11 ng·g^−1^ FW and 5.56 ng·g^−1^ FW, respectively. In root, CK and CJ had significantly higher abscisic acid content at 1.66 ng·g^−1^ FW and 1.62 ng·g^−1^ FW, respectively (Figure 8B). Overall, leaves from PG and JJ treatments accumulated significantly higher levels of aminocyclopropane carboxylic acid and abscisic acid.

#### 2.5.3. Other Hormones

**Figure 9:**
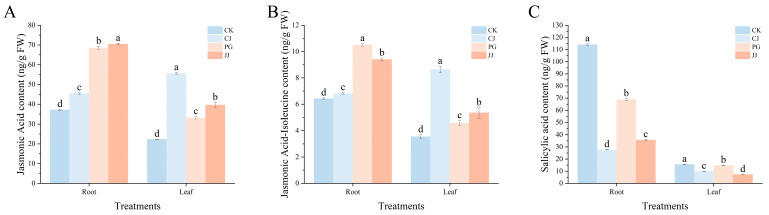
Effects of different interstocks on the other hormone content of tree leaves and roots. (**A**) Jasmonic acid. (**B**) Jasmonic acid-isoleucine. (**C**) Salicylic acid. The parameter values presented in each figure are indicated as mean ± standard deviation (SD) (*n* = 3), different letters denote statistically differences between different treatments (Tukey’s test, *p* < 0.05).

Trends in jasmonic acid and jasmonic acid-isoleucine levels in roots and leaves under different treatments were generally consistent. CJ treatment had the highest content in leaves, while CK treatment showed the lowest content in roots (Figure 9A,B). Salicylic acid content in both the CK roots and leaves was significantly the highest, measuring 114.04 ng·g^−1^ FW and 15.55 ng·g^−1^ FW, respectively. Additionally, the CJ treatment in the roots and the JJ treatment in the leaves exhibited significantly the lowest salicylic acid levels, at 27.63 ng·g^−1^ FW and 7.25 ng·g^−1^ FW, respectively (Figure 9C).

### 2.6. Correlation Analysis

**Figure 10:**
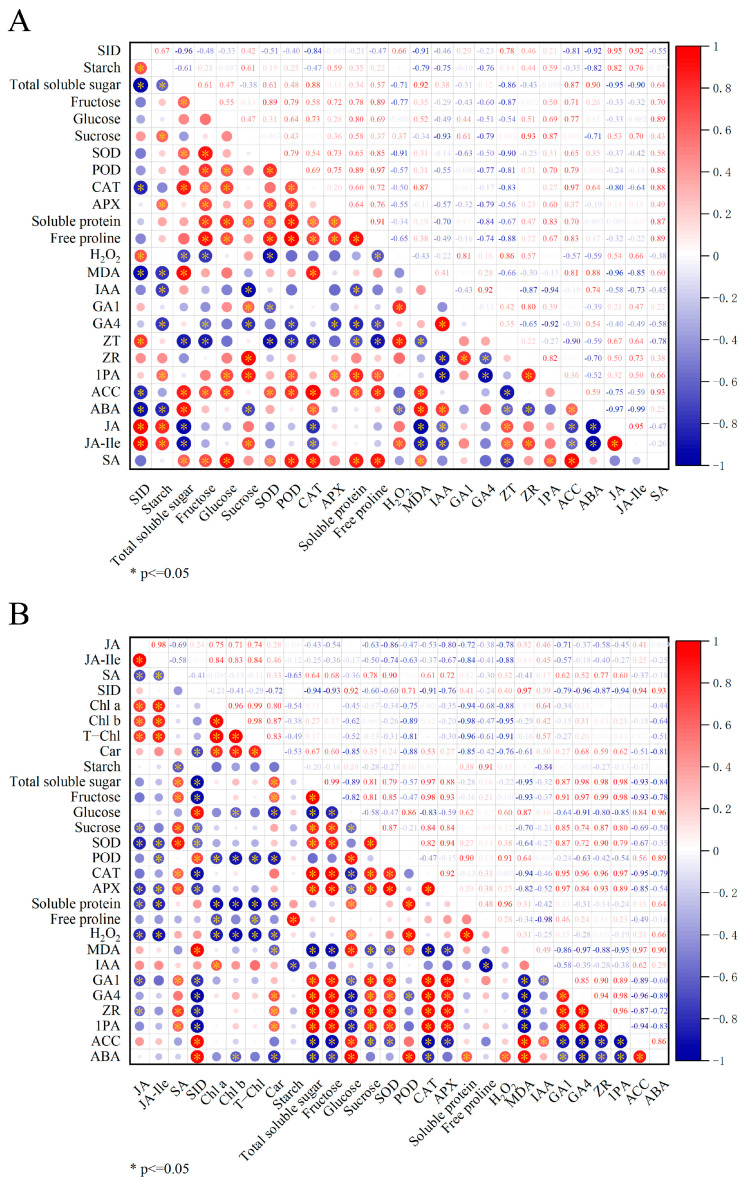
Correlation analysis of each physiological indicator. Note: In the figure ‘SID’ is the abbreviation for ‘scion/interstock diameter’. (**A**) Correlation between SID and various physiological indices of the roots. (**B**) Correlation between SID and various physiological indexes of leaves.

Based on the results presented in Figure 1, a significant difference was found in the ratio of scion diameter to interstock diameter (SID). PG and JJ treatments showed a pronounced phenomenon of “small feet”. To identify physiological indicators related to this ratio, this section will analyze the correlation between SID and various physiological indicators. As shown in Figure 10, the correlation analysis revealed that SID was significantly positively correlated with starch, H_2_O_2_, zeatin, jasmonic acid, and jasmonic acid-isoleucine levels in the roots. Conversely, it was significantly negatively correlated with total soluble sugars, CAT, MDA, aminocyclopropane carboxylic acid, and abscisic acid levels.

In the analysis of leaf physiological indicators, SID showed a significant positive correlation with glucose, POD, MDA, aminocyclopropane carboxylic acid, and abscisic acid, while it was significantly negatively correlated with total soluble sugars, fructose, sucrose, SOD, CAT, APX, and gibberellin A1/A4. These results indicate that there are significant differences roots and leaves responses to SID in grafted plants.

Further analysis revealed that SID is significantly negatively correlated with the total soluble sugars and CAT in both the roots and leaves. Specifically, the correlation coefficients between SID and total soluble sugars and CAT in roots were −0.96 and −0.84 (*p* ≤ 0.05), respectively. In leaves, the correlation coefficients for SID with total soluble sugars and CAT were −0.94 and −0.91 (*p* ≤ 0.05), also indicating significant negative correlations. These results suggest that as SID increases, total soluble sugars and CAT levels in both roots and leaves decrease significantly. Therefore, total soluble sugars or CAT levels in roots or leaves could serve as potential indicators for evaluating grafting incompatibility mediated by interstock in this study.

## 3. Discussion

Several studies have confirmed the influence of rootstock or interstock on tree growth [20,21,22]. Our study similarly observed that the JJ treatment, grafted for 3 years, significantly increased tree height (Figure 1B). This effect could potentially be attributed to the efficient utilization of light and CO_2_ by the leaves of this treatment during the seedling stage [9]. Alongside the JJ treatment, the PG treatment demonstrated comparable performance, suggesting it also enhances rapid tree growth during the seedling stage [9]. However, both treatments exhibited significant “small feet” after 3 years of grafting (Figure 1G). Based on these findings, we consider that the initial rapid growth of the scion in both treatments may directly contribute to the observed “small feet” phenomenon.

As a specific manifestation of grafting incompatibility, “small feet” often result in abnormalities in tree growth, such as yellowing and defoliation of foliage, loss of vigor, growth cessation, or even death [7,12,23]. Previous studies have indicated that graft success correlates positively with leaf chlorophyll content, assessed across various graft combinations [7]. In our study, both the JJ and PG treatments, which displayed graft incompatibility, showed lower chlorophyll content, consistent with prior research findings (Figure 2). The observed differences may be related to blocking carbohydrate assimilation or nitrogen uptake in the leaves of grafted scions from incompatible combinations [24]. Further research on carotenoid content revealed that PG and JJ treatments had significantly lower levels compared to the CK and CJ treatments, especially JJ treatment, which was 15.83% and 20.64% lower than CK and CJ treatments, respectively (Figure 2D). Previous studies have shown that carotenoids perform two main functions in photosynthesis: first, they act as accessory pigments, expanding the wavelength range of light energy utilized in photosynthesis; second, they play a protective role for chlorophyll pigments [25]. The above results indicate that the light harvesting efficiency and the ability to protect chlorophyll in the scion leaves treated with PG and JJ were both inhibited, which may further affect the photosynthetic performance of the leaves, thereby influencing the synthesis and conversion of carbohydrate substances. Currently, there have been reports on the genetic regulation of carotenoid synthesis and degradation. For example, genes involved in synthesis include *PSY*, *PDS*, *ZDS*, *LCYE*, etc., while *CCD4* is the gene that has been clearly reported to participate in carotenoid degradation, mainly by catalyzing the degradation of α-carotene and β-carotene, thus affecting carotenoid accumulation [26]. In relation to this study, the significant decrease in leaf carotenoids under JJ treatment may be associated with differential expression of the aforementioned genes, but this conclusion requires further research and verification.

Moreover, studies have suggested that carbon export from incompatible scions to the rootstock via the phloem is hindered, leading to reduced nitrogen assimilation [27]. Our findings support this notion, as we observed significantly lower levels of soluble sugars in both leaves and roots of the graft incompatibility JJ treatment and PG treatment. Specifically, soluble sugar content in JJ-treated leaves was only 40.67% and 60.10% of that in CK and CJ-treated leaves, respectively (Figure 3B). Sugars, as substrates for respiration, are crucial for energy production. As metabolic intermediates, they are essential for the synthesis of macromolecules [28]. In studies on cucumber and pumpkin grafting, it was found that successful grafting, reconnection of the xylem, and the growth of grafted plants are all promoted by exogenous glucose [29]. Besides glucose, sucrose has also been found to be involved in plant graft compatibility. Guan et al. [28] discovered in their study on cucumber/pumpkin grafting that the difference in sucrose concentration between the rootstock and scion during the early healing stage can affect graft compatibility by altering adhesion at the cutting surface. This indicates that sugars play an important role and function in plant graft compatibility [30]. In this study, the soluble sugar content in leaves and roots of PG and JJ treatments, which exhibited graft incompatibility, was lower compared to CK and CJ treatments. This suggests that graft incompatibility may lead to the suppression of sugar synthesis or accelerated consumption in the leaves and roots of grafted plants. Sugars not only serve as energy sources but also as important signaling molecules, regulating various growth processes and enhancing plant tolerance to both abiotic and biotic stresses [31]. When plants are subjected to stress, sugars can act as osmotic protectants, boosting the plant’s resistance to stress [32,33]. However, the lower sugar content in the leaves and roots of PG and JJ treatments compared to the control group suggests weaker stress resistance in plants. This may be an important reason for the poor vitality observed in graft-incompatible plants at later stages (such as reduced photosynthetic pigments and leaf chlorosis).

To further specify the types of sugars affected, we quantified three sugars−fructose, glucose, and sucrose−finding that changes in fructose and sucrose mirrored the trends in total soluble sugar content in the leaves. This suggests that these sugars are likely the primary components responding to graft incompatibility. Interestingly, we also noted significantly higher starch contents in both roots (2.73%) and leaves (4.51%) of the JJ treatment compared to other treatments (Figure 3A). This aligns with previous observations in other graft-incompatible scenarios, which may be related to the plant’s adaptation to graft-induced stress [14]. Analysis of total starch, total soluble sugars, and three sugar components revealed that the starch content in roots of PG was greater than that in leaves. In addition, fructose and sucrose content in roots under PG and JJ treatments showed a trend that was inconsistent with that in leaves, with root concentrations also higher than those in leaves. This unique observation has not been reported in the literature to date and requires further investigation (Figure 3 and Figure 4).

Antioxidant defense systems play a crucial role in the graft healing process [34,35]. Previous studies on lychee [36], tomato [37], and cucumber [38] have shown that graft combinations with better compatibility exhibit higher enzyme activities such as SOD, POD, APX, and PPO in their leaves. Conversely, combinations with poor graft compatibility tend to show elevated levels of reactive oxygen species or reduced efficiency in their detoxification systems [39]. H_2_O_2_ is one of the most common reactive oxygen species with strong oxidizing properties, and excess reactive oxygen species can lead to lipid peroxidation of cell membranes and damage to DNA and proteins [40].

In our study, both root and leaf enzyme activities were significantly higher in the CK treatment, whereas overall enzyme activities were lower in all interstock treatments, suggesting that interstock intervention may diminish the antioxidant capacity of the tree’s leaf and root systems (Figure 5). Furthermore, CAT activity in leaves was significantly lower in the JJ treatment compared to others, with the lowest levels of SOD and APX activities observed in the leaves. Additionally, the JJ-treated leaves exhibited higher levels of soluble proteins, free proline, H_2_O_2_, and MDA, indicating a reduced detoxification efficiency of the antioxidant system and increased levels of reactive oxygen species (Figure 5 and Figure 6). These observations are characteristic of typical graft incompatibility. Liang et al. [41] found in vegetable grafting studies that grafted plants with stronger antioxidant capacity and lower reactive oxygen species levels had a more active carbon-nitrogen metabolism system, resulting in better growth in later stages. Based on the results of this study, it is speculated that the lower sugar content in JJ treatment may be related to an imbalance in the plant’s antioxidant system. Interestingly, the PG and JJ treatments, which exhibited the “small feet” phenomenon, demonstrated significantly higher POD activities in both leaves (Figure 5B). This finding contrasts with previous studies [36,38]. Research suggests that POD plays a critical role in lignin synthesis and cell wall reinforcement, influencing tissue lignification and promoting vascular bundle formation by modulating hormone levels [42]. Based on these observations, our findings indicate that POD not only participates in graft-induced antioxidant responses but also contributes to various complex physiological processes within the plant.

Plant hormones play important functions and roles in plant growth and development [43]. In the present study we comprehensively characterized the leaf and root hormones of each grafted combination and a total of 11 hormones were detected (Figure 7, Figure 8 and Figure 9). Comparison revealed that the levels of each hormone were similar in the leaves of PG and JJ treatments in grafted combinations. Gibberellin A4 content was lower, and similar results were observed in zeatin-Riboside and N6-(delta2-Isopentenyl) adenosine. In addition, PG and JJ treatments leaves demonstrated significantly higher levels of aminocyclopropane carboxylic acid and abscisic acid. Previous studies have indicated that aminocyclopropane carboxylic acid, as a direct precursor of ethylene, has a direct role in determining the content of ethylene [44,45]. In this study, correlation analysis revealed that SID was significantly and positively correlated with aminocyclopropane carboxylic acid, indicating that aminocyclopropane carboxylic acid is closely related to graft compatibility, although this conclusion requires further research and verification. Both ethylene and abscisic acid have long been recognized as hormones associated with stress and senescence [46,47,48,49]. In pomelo graft studies, it was also found that leaf abscisic acid content was significantly higher in incompatibility combinations, which may be related to the high expression of leaf *NCEDs* induced by grafted incompatibility combinations [14], and similar results were also observed in tomato grafting [50]. The above results suggest that aminocyclopropane carboxylic acid and abscisic acid in leaves can be used as a reference phytohormone for predicting plant graft compatibility in this experiment.

ABA is often closely related to the plant’s antioxidant response. For example, Shu et al. [51] in their study on pumpkin grafting, ABA accumulation promoted H_2_O_2_ generation. Based on the ABA and H_2_O_2_ content in the leaves of PG and JJ treatments in this study, it is suggested that the generation of H_2_O_2_ in leaves may be related to high accumulation of ABA. Additionally, previous studies have found that ABA is also regulated by glucose [52]. Specifically, glucose can promote the activity of enzymes related to ABA biosynthesis, such as zeaxanthin epoxidase (ZEP), 9-cis-epoxy-carotenoid dioxygenase (NCED), and aldehyde oxidase (AO), while reducing the activity of the degradation enzyme ABA 8’-hydroxylase (ABA8’-H), thereby promoting ABA biosynthesis and inhibiting its degradation [51]. In this study, the glucose content in leaves of PG and JJ treatments was significantly higher than that in CK and CJ treatments, which may be another important reason for the significantly higher ABA content in these two treatments.

Overall, in this study, compared to graft-compatible plants, the scion leaves of graft-incompatible plants exhibited lower levels of carotenoids, total soluble sugars, and CAT activity, along with higher levels of H_2_O_2_, MDA, aminocyclopropane carboxylic acid, and abscisic acid. In roots, only the total soluble sugar content showed a trend consistent with that observed in leaves. On the whole, scion leaves are more suitable than roots as research subjects for graft compatibility studies. Among the three interstocks, ‘Harumi’ may be more suitable as the interstock for ‘Yuanxiaochun’. This study only explored the mechanisms of graft incompatibility from a physiological perspective. Future research could further investigate this topic from transcriptional and epigenetic angles to gain a deeper molecular understanding of the specific mechanisms underlying graft incompatibility.

## 4. Materials and Methods

### 4.1. Plant Materials and Treatment

The experiment utilized three-year-old ‘Yuanxiaochun’ grafted seedlings as the experimental material. The interstocks of the grafted seedlings were ‘Harumi’ (*Citrus reticulata* × (*Citrus reticulata* × *Citrus sinensis*)), ‘Ponkan’, and ‘Kumquat’, respectively. The rootstock was ‘*Trifoliate orange*’ (*Poncirus trifoliata* (L.) Raf.). Notably, the control group consisted of ‘Yuanxiaochun’/‘*Trifoliate orange*’, while each grafting combination was represented by CJ, PG, JJ and CK, respectively (Table 1). All grafted seedlings were uniform in condition prior to treatment, with scion diameters measuring between 0.6 and 0.8 cm. These parameters were consistent with those outlined in a previously published study [9]. Each treatment selected 15 grafted seedlings with consistent growth vigor, green leaf color, good graft union healing, and no obvious pests or diseases as experimental subjects, with 5 seedlings in each block and three replicates. All seedlings were managed according to standard horticultural cultivation methods as reported [53]. The experiment was conducted in Zhuangjia Village, Hongyuan Township, Jianyang City, Sichuan Province, China (Longitude: 104°, latitude: 30°). The area is characterized by a subtropical monsoon climate with an average annual temperature of 17 °C, average annual precipitation of 874 mm, and a frost-free period of approximately 311 days per year.

### 4.2. Test Sample Collection and Processing

Based on previous studies [9,11], during the active growth period of citrus (21 May 2022), the growth status of each treatment was recorded, and samples were collected. Healthy functional leaves from the upper parts of the plants in each treatment were selected, and surface stains on the leaves were washed with distilled water and wiped dry. The leaves were then quickly placed on a clean plastic film that had been disinfected with alcohol, with an adequate amount of ice cubes placed underneath the film. The main leaf veins were removed using scissors, and the leaves were then chopped and thoroughly mixed. One portion of the leaves was used to measure chlorophyll content, while the other portion was treated with liquid nitrogen and stored in a −80 °C freezer for future use. Subsequently, the plants were excavated, and the soil within the root system was thoroughly washed with distilled water. The young peripheral roots were then collected, rapidly frozen in liquid nitrogen, and stored at −80 °C for future analysis.

### 4.3. Measurement of Tree Growth Parameters

A tape measure was used to measure the overall height of grafted seedlings for each treatment. A vernier caliper was used to measure the diameter of the rootstock, the diameter of the interstock (at the midpoint), and the diameter of the scion (3 cm above the grafting point).

### 4.4. Determination of Leaf Chlorophyll Content

The determination of chlorophyll content was performed with the method of Molnárová and Fargašová [54]. Accurately weigh 0.50 g of thoroughly mixed leaves, add 10 mL of a mixed extraction solution of acetone and ethanol in a volume ratio of 1:1, seal, and avoid light during an extraction period of 24 h. After extraction, filter the solution, take 200 μL of the filtrate, and measure its absorbance at 663, 645, and 470 nm. Use a mixture of ethanol and acetone as a blank control, and calculate the chlorophyll content. The chlorophyll *a* (Chl *a*) = (12.7D_663_ − 2.69D_645_) × 10/1000W, chlorophyll *b* (Chl *b*) = (22.9D_645_ − 4.68D_663_) × 10/1000W, total chlorophyll (T-Chl) = (20.0D_645_ + 8.02D_663_) × 10/1000W, Carotenoid (Car) = (1000D_470_ − 3.27 Chl *a* − 104 Chl *b*)/229 × 10/1000W.

### 4.5. Determination of Leaf and Root Carbohydrates

Determination of starch and soluble total sugar content was conducted using the anthrone colorimetric method with slight modifications [55]. Precisely weigh 0.25 g of powdered leaf and root samples, place them in 15 mL centrifuge tubes, and add 10 mL of deionized water. Extract in a boiling water bath for 1 h, allow to cool naturally, then centrifuge at 8000 rpm for 3 min. The supernatant is used for soluble total sugar determination. Continue heating 5 mL of distilled water in the precipitate, stirring evenly, and heat in a boiling water bath for 15 min to gelatinize. After removal, add 1 mL of cold 9.2 mol/L perchloric acid to each tube, extract for 15 min, then centrifuge at 8000 rpm for 3 min. The supernatant is used for starch determination.

The determination of fructose, glucose, and sucrose content was conducted using high-performance liquid chromatography (HPLC), the mobile phase consisted of acetonitrile and water in a ratio of 80:20 (*v*/*v*), with a flow rate set at 1.0 mL/min. The column temperature was maintained at 30 °C, and the injection volume was 20 μL [56]. Precisely weigh 1.50 g of leaf and root samples, add 4 mL of distilled water, and shake thoroughly. Further, heat in a water bath at 80 °C for 15 min, then allow to cool naturally. Centrifuge at 9000 rpm at 4 °C for 15 min and collect the supernatant. The residue is extracted again with 4 mL of distilled water, and after combining the supernatants, the volume is adjusted to 10 mL with distilled water. Extracted sample solution (1.50 mL) was withdrawn using a disposable syringe, filtered through a 0.45 μm aqueous phase membrane filter into a vial, and then subjected to HPLC analysis.

### 4.6. Determination of Antioxidant Enzymes and Osmoregulatory Substances in Leaf and Root Systems

After grinding the experimental leaf and root materials with a mill under liquid nitrogen protection, 0.50 g of the sample was weighed using an electronic balance (AL204, METTLER, Greifensee, Switzerland) and added to a 10 mL centrifuge tube. Then, 5 mL of pH 7.80 pre-chilled phosphate buffer solution were added and thoroughly mixed. The mixture was centrifuged at 4 °C for 20 min (10,000× *g*), and the supernatant (enzyme solution) was carefully collected and its total volume (V) measured. The collected supernatant was transferred into clean tubes and stored at 0–4 °C for further use. The determination of superoxide dismutase (SOD), peroxidase (POD), catalase (CAT), ascorbate peroxidase (APX), soluble proteins, and malondialdehyde (MDA) followed the methods outlined by Cao Jiankang et al. [55], with slight modifications.

#### 4.6.1. SOD Activity

The activity of SOD was measured using the nitroblue tetrazolium (NBT) method. Three 10 mL test tubes of the same model were selected and labeled as 1, 2, and 3, with tube 1 serving as the experimental tube and tubes 2 and 3 as control tubes. Each tube received 3 mL of SOD reaction solution, and 50 μL of enzyme solution was added to tube 1 and mixed thoroughly. Tubes 2 and 3 were filled with phosphate buffer solution instead, mixed well, and tube 3 was placed in the dark. The other two tubes were exposed to light at 4000 Lux for 30 min. After the reaction, the absorbance of each tube was measured at a wavelength of 560 nm, using the dark control tube as a blank for calibration, and the SOD activity was subsequently calculated.

#### 4.6.2. POD Activity

The activity of POD was measured using the guaiacol colorimetric method. A volume of 10 μL of each treated enzyme solution was added to 1.50 mL of POD reaction solution and mixed thoroughly. Following this, 200 μL of the mixed solution was rapidly transferred to a spectrophotometer to measure the absorbance at 470 nm. Readings were taken every 30 s for a total of five measurements, with the enzyme activity represented by the change in absorbance per minute.

#### 4.6.3. CAT Activity

CAT activity was measured using the ultraviolet absorption method. An accurate volume of 5.0 μL of enzyme solution was added to 295 μL of CAT reaction solution and mixed thoroughly. Following this, 200 μL of the mixed solution was rapidly transferred to a spectrophotometer for measurement at 240 nm. Readings were taken every 30 s for a total of five measurements. The change in absorbance at 240 nm per minute, denoted as ΔOD240, was calculated, with one unit of catalase activity defined as the increase of 0.01 in absorbance per gram of fresh weight (FW) of the sample per minute.

#### 4.6.4. APX Activity

APX activity was measured using the ascorbic acid (ASA) oxidation method. An accurate volume of 100 μL of enzyme solution was added to 2900 μL of APX reaction solution and mixed thoroughly. Following this, 200 μL of the mixed solution was rapidly transferred to a spectrophotometer for measurement at 290 nm. The changes in absorbance were recorded over a time span of 10 to 40 s to calculate the enzyme activity.

#### 4.6.5. Soluble Protein Content

The soluble protein content was measured using the coomassie brilliant blue G-250 dye-binding method. An accurate volume of 250 μL of the enzyme solution was mixed with 2.5 mL of the G-250 reaction solution and allowed to stand for 2 min. The absorbance was then measured at 595 nm. The soluble protein content of the sample was calculated based on a standard curve prepared from known protein concentrations.

#### 4.6.6. MDA Content

MDA content was measured using the thiobarbituric acid (TBA) method. An accurate volume of 1 mL of enzyme solution was mixed with 2 mL of 0.6% TBA solution. The mixture was sealed and incubated in a boiling water bath for 15 min. Afterward, it was rapidly cooled and centrifuged to obtain the supernatant. The absorbance of the supernatant was measured at three wavelengths: 600 nm, 532 nm, and 450 nm. The MDA content was then calculated based on the absorbance values obtained.

### 4.7. Determination of Free Proline and Hydrogen Peroxide (H_2_O_2_)

The content of free proline was determined using the acid ninhydrin method [55]. Exactly 0.30 g of the sample was weighed and added to 3.0 mL of salicylic sulfonic acid, followed by heating in a water bath at 93 °C for 10 min. After cooling, the mixture was centrifuged at 20 °C for 10 min at 10,000 rpm. The supernatant was collected, added to the reaction solution, and then 200 μL was measured for absorbance at 520 nm. The content was calculated based on a standard curve. H_2_O_2_ was determined using the titanium tetrachloride colorimetric method [55]. Exactly 1.0 g of pulverized sample was weighed and added to 5.0 mL of pre-chilled acetone, mixed thoroughly, and then centrifuged at 12,000× *g* and 4 °C for 20 min. The supernatant was collected. The supernatant was aspirated, added to the reaction solution, centrifuged, and the supernatant was discarded, leaving the precipitate. The precipitate was then treated with acetone to remove pigment. Finally, 3.0 mL of 2 mol/L sulfuric acid was added to the washed precipitate. After complete dissolution, colorimetric analysis was performed, and the content was calculated based on a standard curve.

### 4.8. Determination of Leaf and Root Hormones

The hormones in the tree’s roots and leaves were analyzed by Shanghai Sanshu Biological Technology Co., Ltd. (Shanghai, China). using liquid chromatography-high-resolution mass spectrometry. Accurately weigh 100 mg of the sample and add 1.0 mL of pre-cooled 50% acetonitrile (ACN) aqueous solution. Subsequently, subject the sample to ultrasonication at 4 °C for 3 min, followed by extraction at 4 °C for 30 min. After extraction, centrifuge the sample at 12,000 rpm for 10 min at 4 °C, and collect the supernatant for further processing and analysis. The results were based on high-resolution mass spectrometry, with qualitative analysis conducted through the identification of secondary mass spectrometry fragments. Quantitative analysis was performed using the external standard method.

### 4.9. Statistical Analysis

Statistical significance was evaluated using analysis of variance (ANOVA) in SPSS 23.0 (IBM, Armonk, NY, USA), with a significance threshold set at *p* < 0.05. Origin 2021 (OriginLab Corporation, Northampton, MA, USA) software was used to draw the figures.

## 5. Conclusions

In our study, graft incompatibility was observed when using ‘*Trifoliate orange*’ as rootstock and ‘Ponkan’ and ‘Kumquat’ as interstocks grafted on ‘Yuanxiaochun’. The incompatible graft combinations exhibited several distinct characteristics: (1) The carotenoid content in the leaves and total soluble sugars in leaves and roots were significantly reduced. (2) The CAT activity in the leaves decreased, while the levels of H_2_O_2_ and MDA increased. (3) Lower concentrations of leaf gibberellin A4, zeatin-Riboside, and N6-(delta2-Isopentenyl) adenosine were reported, while significantly elevated levels of aminocyclopropane carboxylic acid and abscisic acid were observed. This study comprehensively analyzed physiological and biochemical parameters in the roots and leaves of graft-compatible and incompatible combinations. Our findings suggest that indicators such as carotenoids, total soluble sugars, CAT activity, and abscisic acid content in leaves are critical markers for evaluating graft compatibility. These markers can be used to assess the early compatibility of graft combinations, providing a more accurate and efficient method for selecting those with higher compatibility. Furthermore, these biochemical parameters can serve as key tools in the early-stage evaluation of grafting success, offering valuable insights for practical applications. By utilizing these markers, growers can optimize the selection of interstocks, thereby improving grafting success rates and enhancing overall citrus production efficiency.

## Figures and Tables

**Table 1:** Test material treatment and abbreviations.

Treatments	Abbreviations
‘Yuanxiaochun’/‘*Trifoliate orange*’	CK
‘Yuanxiaochun’/‘Harumi’/‘*Trifoliate orange*’	CJ
‘Yuanxiaochun’/‘Ponkan’/‘*Trifoliate orange*’	PG
‘Yuanxiaochun’/‘Kumquat’/‘*Trifoliate orange*’	JJ

## Data Availability

Data are contained within the article.
